# BN-Doped Polycyclic Aromatic Hydrocarbons and Their Applications in Optoelectronics

**DOI:** 10.3390/molecules30214252

**Published:** 2025-10-31

**Authors:** Liping Jia, Qiuhuan Wu, Teng Yang, Binghui Xie, Jie Sheng, Wucheng Xie, Junjun Shi

**Affiliations:** College of Environment and Chemical Engineering, Foshan University, Foshan 528000, China; jialiping@fosu.edu.cn (L.J.); 2112460046@stu.fosu.edu.cn (Q.W.); yangteng@fosu.edu.cn (T.Y.); 2112460048@stu.fosu.edu.cn (B.X.); s498952506@163.com (J.S.)

**Keywords:** BN-doped polycyclic aromatic hydrocarbons, optoelectronic applications, OLED, OFET, photophysical properties, electrical properties, synthetic strategy

## Abstract

This article reviews the research progress of BN-doped polycyclic aromatic hydrocarbons in the field of optoelectronics in recent years. Polycyclic aromatic hydrocarbons and their derivatives have attracted widespread attention in the field of organic optoelectronic materials due to their unique optical and electronic properties and thus are widely used in various fields such as organic light-emitting diodes (OLEDs), field-effect transistors (OFETs), and organic solar cells (OSCs). By introducing boron–nitrogen (BN) units to replace carbon–carbon (C-C) units in the framework of polycyclic aromatic hydrocarbons, the electronic structure and spatial configuration of conjugated molecules can be effectively regulated, thereby optimizing their optoelectronic properties. This article first outlines the structural characteristics of BN-doped polycyclic aromatic hydrocarbons, then explores their synthesis methods and properties, and provides a detailed overview of their applications in the field of optoelectronics. BN-doped polycyclic aromatic hydrocarbons have shown great potential for applications in both optical and electrical fields. Finally, this review points out that although the application of BN-embedded polycyclic aromatic hydrocarbons in optoelectronic devices is still in its early stages, facing difficulties in synthesis and insufficient stability, the rational design of BN to replace polycyclic aromatic hydrocarbons is expected to bring new opportunities for organic electronics and advance the development of this field.

## 1. Introduction

Organic semiconductor materials are increasingly becoming a strong competitor in the new generation of electronic component materials due to their significant advantages such as easy structural diversification and tunability, light weight, low cost, excellent flexibility, and the ability to enable large-area film fabrication under low-temperature conditions. They are widely used in cutting-edge fields such as organic field-effect transistors (OFETs) [1], organic solar cells (OPVs) [2], organic light-emitting diodes (OLEDs), and sensors, and have attracted extensive attention from the scientific community [3]. In the current context, it is particularly important to design and synthesize organic semiconductor materials with novel structures in order to significantly improve the performance of organic electronic devices. These materials are mainly composed of small molecules or polymers containing π-conjugated structures, which provide strong impetus for the vigorous development of organic electronics at the source. By introducing heteroatoms such as sulfur, nitrogen, and oxygen into the conjugated carbon skeleton, these conjugated molecules exhibit diverse photophysical and electronic properties, greatly enriching their application potential. By finely regulating the molecular structure and interactions between them, we can effectively change the electronic structure and spatial configuration of the material [4,5]. Heteroatoms in aromatic systems also induce intramolecular/intermolecular interactions (e.g., hydrogen bonding, S-S interactions) that influence solid-state packing and device performance [6,7]. Notably, BN units—isolectronic with C-C units—offer a powerful strategy for tuning polycyclic aromatic semiconductors. Replacing C-C with B-N units in polycyclic aromatics enables broader performance optimization, so we are able to explore and optimize the performance of organic materials in a broader scope, providing infinite possibilities for innovative applications of organic semiconductor materials.

Polycyclic aromatic hydrocarbons (PAHs), which are aromatic polycyclic compounds formed by fusing multiple benzene rings, have shown wide application value in the field of organic photovoltaics [8,9,10,11]. In recent years, many researchers have shown great interest in doping heteroatoms (such as B, N, O, Si, P, S) into the π-conjugated framework of polycyclic aromatic hydrocarbons (PAHs) [12]. This doping strategy not only greatly enriches the structural diversity of the π-conjugated system of PAHs, but also brings unprecedented photophysical properties, further expanding the diversified choices for molecular design of organic optoelectronic materials. In 1958, Dewar and his colleagues conducted groundbreaking research on BN-substituted polycyclic aromatic hydrocarbons (PAHs), exploring for the first time the issue of one or more B-N fragments replacing C-C fragments in classical aromatic hydrocarbons. This research achievement has laid a solid foundation for progress in the field of nitrogen hybridization chemistry [13]. Subsequently, numerous BN-embedded aromatic hydrocarbons were synthesized one after another. Due to the complexity of the synthesis and purification process of nitrogen-containing heterocyclic compounds, early research mainly focused on exploring synthesis methods and characterizing chemical properties [14]. With the continuous progress in the field of nitrogen hybrid chemistry, the potential applications of BN-embedded aromatic hydrocarbons in various fields such as biological systems, coordination chemistry, and materials science are becoming increasingly prominent, which has aroused widespread attention to this class of novel molecules. More importantly, the introduction of BN units has brought new optical and electronic properties to polycyclic aromatic hydrocarbons, opening up new avenues for the application prospects in the field of optoelectronic devices [15]. However, despite the enormous potential of BN-embedded aromatic hydrocarbons, the exploration of their optoelectronic applications is still in its infancy, and this field is attracting increasing attention and investment from chemists and materials scientists [16]. Given the widespread presence of C-C double bonds in organic π-conjugated systems, this article focuses on the isoelectronic relationship between B-N single bonds and C-C double bonds and reviews the latest application progress of BN compounds in optoelectronic fields such as light-emitting devices, photodetectors, photocatalytic materials, field-effect transistors, photovoltaic devices, and electrochemical sensors. At the end of the article, the current status of the application of BN compounds in optoelectronics is analyzed and summarized, and prospects for the future application of BN compounds in more fields are discussed.

## 2. An Overview of the Structure of BN-Doped Polycyclic Aromatic Hydrocarbons

In 1919, Langmuir first proposed the concept of electronic isostere in the field of inorganic chemistry. This concept defines a special class of molecules, atoms, or groups (ions) that have the same number of atoms and electrons, and the arrangement of electrons remains consistent. These are considered electronic isosteres. Examples such as N_2_ and CO, N_2_O and CO_2_, and N_3_^−^ and NCO^−^ all belong to this category and exhibit similar properties. Similarly, benzene, thiophene, and pyridine are also regarded as examples of electron isostructures due to their similar physicochemical properties [17]. C=C has a higher bond enthalpy due to the strong overlap of non-polar and π bonds; B=N has a lower bond energy due to its polarity, but its geometric parameters (bond length, bond angle) are similar to those of C=C. This is also one of the reasons why boron nitride compounds are often referred to as “inorganic benzene” and “isoelectronic analogues” [18,19,20]. In 1958, Dewar’s research group pioneered the use of a B=N bond to replace the C=C double bond in classical aromatic hydrocarbons, successfully constructing boron-nitrogen hybrid polycyclic aromatic hydrocarbons (BN-PAHs) [13]. In the following decades, the research group continued to work hard and synthesized various BN-PAHs [21]. In recent years, polycyclic aromatic hydrocarbons and their derivatives have attracted widespread attention from researchers in the field of organic optoelectronic materials due to their unique optical and electronic properties. This type of material is particularly prominent in the application of electronic devices, widely used in multiple fields such as organic light-emitting diodes (OLEDs), field-effect transistors (OFETs), and organic solar cells (OSCs) [22,23]. Compared to inorganic materials, organic optoelectronic materials have higher structural plasticity at the molecular level, and by precisely adjusting their structure, they can meet specific performance requirements. Embedding heteroatoms into the framework of all carbon polycyclic aromatic hydrocarbons can effectively regulate their electronic structure and optimize their photophysical properties [24]. On this basis, the concept of electronic isosteres provides a solid theoretical basis for finely adjusting molecular structures. By utilizing the properties of electronic isosteres, molecular structures can be precisely adjusted to meet specific application requirements without changing the number of electrons and spatially related chemical structures. This theory not only promotes the construction of new compounds but also opens new avenues for applications in fields such as biomedicine and functional materials [25].

**Figure 1:**
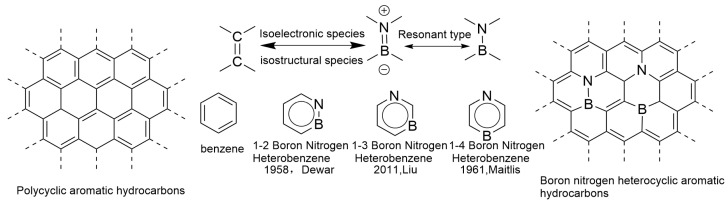
Polycyclic aromatic hydrocarbons and boron–nitrogen heterocyclic aromatic hydrocarbons [26]. Reprinted from Ref. [26].

In recent years, research on boron–nitrogen/carbon–carbon (BN/C-C) electronic emitters has made rapid progress. Due to the fact that boron atoms have three valence electrons in their outermost layer, nitrogen atoms have five valence electrons in their outermost layer, and carbon atoms have four valence electrons in their outermost layer, a C=C unit has exactly eight valence electrons, which is the same number of valence electrons as the boron–nitrogen unit (i.e., the three valence electrons of boron and the five valence electrons of nitrogen) (both have eight valence electrons). Therefore, BN units and C=C units are not only isoelectronic but also isostructural. Replacing the two carbon atoms on the benzene ring with boron and nitrogen atoms can generate a boron–nitrogen heterocyclic aromatic ring analogous to benzene. This compound is not only an isoelectronic and isostructural form of the benzene ring, but also forms three isomers, 1,2-borazepenzene, 1,3-borazepenzene, and 1,4-borazepenzene, based on the relative positions of boron and nitrogen atoms on the benzene ring (Figure 1) [26]. Among them, the 1,2-isomer is the most stable, while the 1,3-isomer is the least stable. The 1,3-arrangement of B and N atoms in the p-electron system results in charge separation, which has been found to be the reason for the lowest stability of the 1,3-isomer. This charge separation, in turn, can be considered the strongest driving force for ring electron delocalization, making the same isomer the most aromatic. Although it is well known that BN bonds weaken electron delocalization due to significant differences in electronegativity between atoms, the 1,4-BN relationship reduces aromaticity to a greater extent by making p-electron delocalization more unidirectional (from N to B). Therefore, 1,4-Azabolin was found to be the least aromatic. Its lower stability compared to 1,2-isomers can be explained by the greater exchange repulsion [27]. In addition, the structural diversity of BN-PAHs has been further expanded, thanks to the unique coordination modes exhibited by boron atoms, namely triple coordination and quadruple coordination.

## 3. An Overview of the Synthesis of BN-Doped Polycyclic Aromatic Hydrocarbons

Introducing main group elements into π-conjugated carbon frameworks has become a highly promising and effective strategy in exploring polycyclic aromatic hydrocarbons with better photophysical and electronic properties. By selecting non-carbon dopants, the frontier orbital energy levels can be precisely controlled, thereby successfully achieving the desired photophysical properties in the target material. Among them, boron and nitrogen have attracted more research attention compared to other potential dopants due to their unique electronic and optical properties [28,29,30,31,32]. It is particularly noteworthy that there exists an isomorphic/isoelectronic relationship between the C-C unit and the B-N unit, which enables the B-N bond to serve as an effective alternative to the C-C bond in polycyclic aromatic hydrocarbons [33,34]. Based on this, numerous BN-doped polycyclic aromatic hydrocarbons have been successfully developed and demonstrated great potential as candidate materials with broad prospects for various optoelectronic applications [35]. However, the optoelectronic application of embedding BN into aromatic hydrocarbons is still in its early stages, and this field is gradually attracting strong interest from chemists and materials scientists.

**Figure 2:**
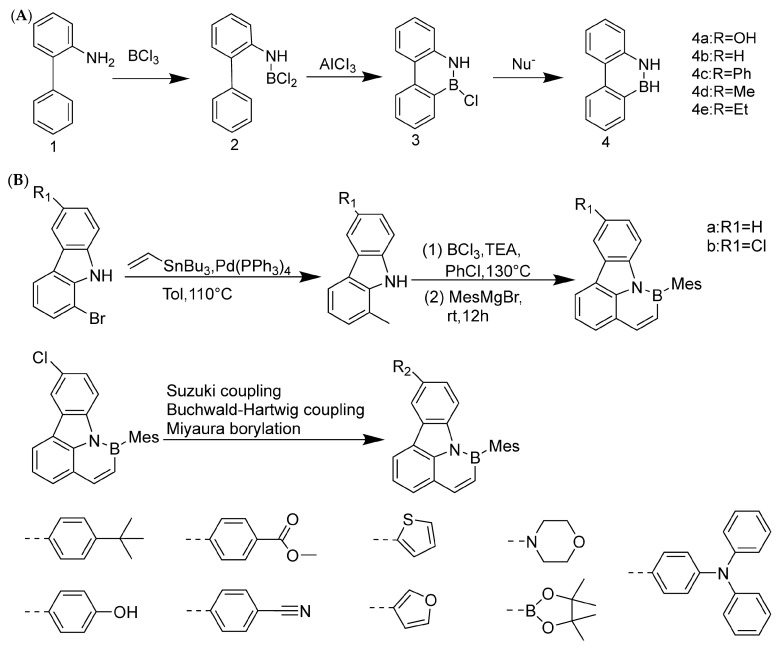
(**A**) Construction of boron–nitrogen heterocycles via electrophilic substitution reaction [13]. Reprinted with permission from Ref. [13]. Copyright 1958 Royal Society of Chemistry (RSC). (**B**) Synthesis of boron–nitrogen heterofluoranthene [43]. Reprinted with permission from Ref. [43]. Copyright 2022 American Chemical Society.

The synthesis history of boron–nitrogen heterocyclic aromatic compounds can be traced back to 1958. Dewar et al. were the first to make a breakthrough in this field (Figure 2A), and they successfully synthesized 5,6-boron-nitrogen phenanthrene through an aromatic electrophilic substitution reaction [13]. In 1959, Dewar’s group extended this work by preparing borazanaphthalene through electrophilic cyclization of o-vinylaniline with BCl_3_, with yields ranging from 40% to 69% for key derivatives (e.g., 2-phenyl-2,1-borazaronaphthalene) [36]. However, poor functional group tolerance and scalability restricted its application to complex PAHs.

Polycyclic aromatic hydrocarbons (PAHs) have shown extensive potential in the field of organic electronics, and the embedding, burial sites, and orientation of BN units have a profound impact on their structure and properties [37,38,39,40]. In view of this, researchers are committed to the precise synthesis of polycyclic aromatic hydrocarbons with specific BN unit orientations [41]. In 2021, based on the synthesis strategy of naphthalene, Pei‘s group successfully developed the first class of parent B_2_N_2_ cyclohexene with different BN orientations. By utilizing known BN-embedded molecules as building blocks and combining them with commonly used synthesis methods in polycyclic aromatic hydrocarbon chemistry, various B_2_N_2_ cycloalkenes with different BN orientations were effectively synthesized. Specifically, by using known BN-embedded molecules as building blocks and integrating standard PAH synthetic methods (e.g., cyclization, cross-coupling), they efficiently synthesized three types of B_2_N_2_-perylenes with yields ranging from 28% to 82%: NBBN-P: synthesized via Yamamoto coupling of dibrominated BN-naphthalene, yielding 72%; BNNB-P: obtained through oxidative coupling and radical cyclization, with an overall yield of 28%; BNBN-P: prepared via Suzuki coupling followed by an intramolecular Heck reaction, achieving an 82% yield for the final step. These three types of B_2_N_2_ peroxymethane were synthesized and characterized with a carbon analogue, peroxymethane. The research results reveal that the doping of BN can effectively regulate (anti-) aromaticity, while the dipole–eddipole interaction between molecules significantly shortens the π-π stacking distance. In addition, the introduction of BN units also changed the distribution of electrostatic potential and solid-state filling mode, presenting a completely new crystalline phase that had not been observed in toluene. Although there are slight differences in the energy levels and frontier molecular orbital distributions of these B_2_N_2_ perpropane molecules, their photophysical properties, such as absorption rate and photoluminescence quantum yield, exhibit significant differences. This work not only provides systematic insights into the effects of BN doping, but also validates an effective synthesis strategy based on embedding more polycyclic aromatic hydrocarbons into existing BN-containing molecules [42].

In 2022, Liu’s group at Tianjin University of Technology achieved an important milestone. They used pyrrole-type nitrogen atoms as a guide and successfully synthesized boron–nitrogen heterofluoranthene through an electrophilic boronation reaction (Figure 2B). DFT calculations showed that the HOMO-LUMO gap of boron–nitrogen fluoranthene was wider than that of all carbon fluoranthene. NICS (Nucleus-Independent Chemical Shift) calculations indicate that the introduction of boron–nitrogen units results in weak aromaticity of the five-membered ring in the fluoranthene structure, while the corresponding five-membered ring in all-carbon fluoranthene is anti-aromatic. This suggests that the introduction of boron–nitrogen units can achieve property regulation of all-carbon compounds. To study the reaction performance of boron–nitrogen fluoranthene, a series of coupling reactions were carried out on boron–nitrogen fluoranthene (**81b**) with an R1 group as the chlorine atom, resulting in eleven derivatives. The study showed that substituents can effectively regulate the photophysical properties of boron–nitrogen heterofluoranthene [43].

In 2023, Li’s group synthesized boron–nitrogen heterocyclic R-ANH and studied the aromatic electrophilic bromination reaction of boron–nitrogen heterocyclic Mes-ANH. Furthermore, the regioselectivity of the bromination reaction was explained through DFT theoretical calculations. After confirming that its bromination position preferentially occurs adjacent to the boron atom, a series of reactions including electrophilic bromination, the Sonogashira coupling reaction, an enyne cyclization reaction, and oxidative dehydrogenation were carried out using the boron–nitrogen heterocyclic compound ViANH as a substrate to obtain the boron–nitrogen heterocyclic compound BNAN and the boron–nitrogen heterocyclic compound BNANH. Optical studies have shown that BNAN, BNANH, and R-ANH all have high quantum yields, and the absorption and emission spectra of BNAN exhibit a certain degree of red-shift compared to BNANH and R-ANH. This study greatly enriches the variety library of boron–nitrogen-doped CP-PAHs and lays the molecular foundation for the subsequent application development of boron–nitrogen-doped benzo [44].

**Figure 3:**
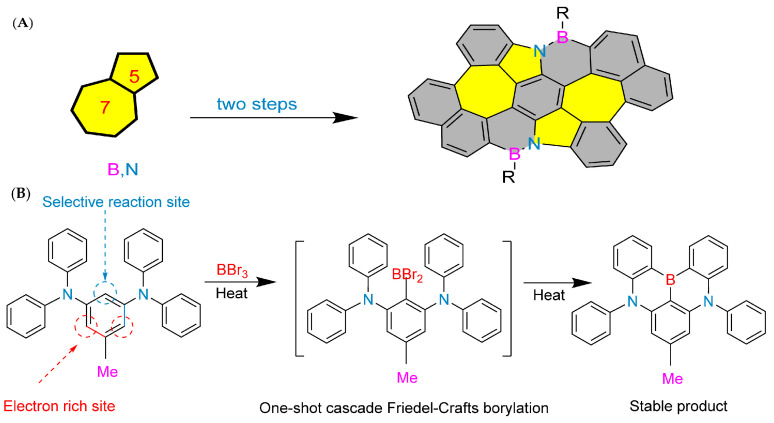
(**A**) BN-2 [45]. Reprinted with permission from Ref. [45]. Copyright 2023 John Wiley and Sons. (**B**) Synthesis of 1,4-BN-doped MR emitters via one-shot borylation using sole boron bromid [46]. Reprinted with permission from Ref. [46].Copyright 2024 John Wiley and Sons.

In 2023, the research team led by Liu’s group published a study introducing highly efficient synthesis of a novel polycyclic aromatic hydrocarbon (BN-2) containing two pairs of pentagonal and heptagonal rings and B_2_N_2_. Using commercially available starting materials, BN-2 was synthesized on a large scale in just two steps (Figure 3A). The synthesized B_2_N_2_-doped molecule (BN-2) exhibited strong green fluorescence, with pentagonal and heptagonal rings, and an absolute photoluminescence quantum yield (ΦBN-2) of 23.1%. Time resolved spectroscopy revealed the contribution of BN-2’s long-lived delayed fluorescence to total fluorescence in the microsecond time range (τ = 19 µs). DFT and TDDFT calculations showed a small ∆ES-T energy separation (0.01 eV) between singlet and triplet excited states, leading to a relatively effective intersystem crossing (ISC) process that can compete with fluorescence radiation attenuation. The measurement technique of flash photolysis and field-induced time-resolved microwave conductivity (FP/FI-TRMC) was used to confirm that BN-2 has an excellent intrinsic carrier mobility of up to 0.03 cm^2^ V^−1^ s^−1^. These properties make it a promising material for optoelectronic devices [45].

Developing organic electroluminescent materials and devices with narrow spectral emission is key to meeting future high-definition display requirements and maintaining the competitiveness of organic light-emitting diode (OLED) technology. Boron/nitrogen (B/N)-based multiple resonance (MR) luminescent materials have become a research hotspot in the field of organic electroluminescence due to their rigid skeleton and unique short-range charge transfer characteristics, which exhibit narrow spectral emission and high efficiency advantages. In February 2024, Yang’s group developed an efficient synthesis method for 1,4-BN-doped polycyclic aromatic hydrocarbons (Figure 3B), making sustainable production of narrowband organic luminescent materials possible. Significant regioselective boronization was achieved by strategically introducing substituents such as methyl, tert-butyl, phenyl, and chloride at the C5 position of the 1,3-phenylenediamine substrate. This method facilitates the synthesis of 1,4-BN-doped polycyclic aromatic hydrocarbons in different ranges, with good yields and excellent regioselectivity. This synthesis method has demonstrated good scalability for large-scale production and achieved post-functionalization of boronized products. The mechanism study provides valuable insights into the key roles of electronic effects and steric hindrance effects in achieving efficient regioselective boronization reactions. In addition, the excellent device performance of the synthesized compounds **10b** and **6z** highlights the practicality and significance of the developed method [46].

In May 2023, Zhou’s group explored and successfully implemented two novel synthetic pathways for BN-doped pyrene derivatives. The first approach focuses on utilizing the photothermal conversion properties of four coordinated boron–nitrogen compounds to achieve the synthesis of boron–nitrogen-doped pyrene-based compounds. The second approach is to use benzoquinoline as a synthetic platform and successfully prepare a single boron–nitrogen-doped pyrene derivative BNP through a boron–silicon exchange reaction between boron tribromide and alkyl silicon. It is worth noting that this reaction represents the first application of the exchange reaction between boron and alkyl silicon, which not only enriches the synthesis methodology of boron–nitrogen-doped pyrene derivatives but also provides another highly promising pathway for the exploration of the synthesis of related compounds in the future [47].

Overall, modern BN-PAH synthesis leverages green chemistry, catalytic methodologies, and modular strategies to achieve efficient, scalable, and sustainable production of structurally diverse and functionally tunable BN-doped polycyclic aromatic hydrocarbons.

## 4. An Overview of the Properties of BN-Doped Polycyclic Aromatic Hydrocarbons

In recent years, the introduction of heteroatoms has not only greatly enriched the structural diversity of π-conjugated systems of polycyclic aromatic hydrocarbons (PAHs), but also endowed them with novel photophysical properties, further expanding the range of choices for molecular design of organic optoelectronic materials. When the C-C units in the conjugated skeleton are replaced with BN units, but also due to the electronegativity difference between boron (electronegativity 2.04) and nitrogen (electronegativity 3.04), the formed BN units exhibit unique dipole moments and ionic characteristics [48,49]. This characteristic enables the regulation of the distribution of frontier molecular orbitals and the interactions between molecules in bulk, thereby profoundly affecting the electronic structure, optical properties, and assembly behavior of organic conjugated systems. With the continuous optimization of scientific research conditions and the discovery of unique optoelectronic properties of BN-PAHs (boron-nitrogen doped polycyclic aromatic hydrocarbons), BN-PAHs have become a research focus for domestic and foreign researchers. Compared to all-carbon aromatic hydrocarbons, some boron–nitrogen aromatic hydrocarbons exhibit a series of unique properties, such as BN bond dipole-induced head-to-tail stacking mode, shorter π-π stacking distance, and higher luminescence quantum yield. These characteristics translate to such hydrocarbons attracting much attention as a novel organic optoelectronic material-based molecule for BN-PAHs [20]. Specifically, BN-PAHs have demonstrated excellent performance in fields such as organic field-effect transistors (OFETs) and organic light-emitting diodes (OLEDs) [19]. For example, studies have shown that in BN-PAHs where boron and nitrogen atoms are not directly connected, boron and nitrogen atoms can induce multiple resonances, resulting in HOMO-LUMO (highest occupied molecular orbital and lowest unoccupied molecular orbital) separation [50], In addition, DABNA-2, as an OLED device with a light-emitting layer, has an external quantum efficiency (EQE) of up to 20.2%, an emission peak full width at half maximum (FWHM) of only 28 nm, and CIE coordinates of (0.12, 0.13), which exhibits narrowband emission of thermally activated delayed fluorescence (TADF) characteristics [51]. These findings further demonstrate the enormous potential and application prospects of BN-PAHs in the field of organic optoelectronic materials.

From the perspective of molecular engineering, the overall dipole properties of BN-PAHs (boron–nitrogen-doped polycyclic aromatic hydrocarbons) can be regulated by adjusting the number, position, and orientation of BN units, thereby endowing BN-PAHs with diverse photophysical properties [52]. Specifically, Zhang Chen’s research in 2021 pointed out that the introduction of boron–nitrogen bonds and their relative positions in space have a significant impact on the photophysical properties of boron–nitrogen-doped phenanthrene. In this study, both types of synthesized boron–nitrogen phenanthrene exhibited certain aromatic characteristics, and these two types of phenanthrene showed different regioselectivity in aromatic electrophilic substitution reactions [53]. In 2025, the Huo research group introduced boron atoms at two different sites on the same aromatic skeleton to synthesize two boron-doped polycyclic aromatic hydrocarbon (PAH) positional isomers, NAPB and BBCZ. Single-crystal X-ray diffraction and DFT calculations indicate that the fusion of boron and naphthalene ring in NAPB induces charge transfer excited states, with an emission peak at 502 nm, a half peak width of 31 nm, and a radiation rate constant of 1.73 × 10^7^ s ^−1^. Meanwhile, the peripheral boron atoms in BBCZ retain local excited state characteristics and emit sky blue light. Research has shown that the site-specific introduction of boron can finely regulate the emission wavelength, bandwidth, and exciton dynamics, providing a convenient strategy for designing narrowband efficient luminescent materials [54].

Taking the photophysical properties of boron–nitrogen-doped benzene as an example, in 2023, Li Wenlong compared the photophysical properties of boron–nitrogen-doped benzene R-ANH and all-carbon benzene C-ANH and found that compared with C-ANH (λabs = 291 nm, λem = 324 nm), R-ANH had a significant red shift in absorption (Mes-ANH: λabs = 316 nm; Ph-ANH: λabs = 324 nm; Vi-ANH: λabs = 323 nm) and emission spectra (Mes-ANH: λem = 359 nm; Ph-ANH: λem = 367 nm; Vi-ANH: λem = 365 nm). This is consistent with the theoretically calculated result showing a decrease in the HOMO-LUMO energy level difference of boron–nitrogen heterocyclic compounds, which once again strongly proves that boron–nitrogen doping has a significant impact on the photophysical properties of polycyclic aromatic hydrocarbons [44].

However, the operational stability of BN-doped polycyclic aromatic hydrocarbons under practical conditions remains a challenge. Their thermal and photochemical instability in operating environments, due to prolonged exposure to light, electrical stress, and environmental conditions, may limit their long-term performance. For example, some BN-doped systems may be prone to oxidation or degradation under irradiation, which may hinder their application in commercial devices. Although BN PAHs typically have higher air and chemical stability than their all-carbon analogues, their stability under strong light, prolonged heating, or the presence of reactive species varies depending on their specific molecular structure and substitution patterns.

In summary, although the direct impact of replacing C-C bonds with BN bonds in polycyclic aromatic hydrocarbons (PAHs) on their structure is relatively small [55], BN-PAHs exhibit unique properties that are completely different from their all-carbon structure [56]. Compared with pure carbon frameworks, BN-PAHs exhibit numerous novel and unique performance characteristics. BN-PAHs are increasingly receiving widespread attention in the field of organic optoelectronic materials and devices due to their more easily adjustable energy levels and bandgaps, stronger intermolecular interactions, higher luminescence quantum efficiency, and superior air stability. They have shown preliminary application potential in multiple fields such as OLEDs (organic light-emitting diodes), OFETs (organic field-effect transistors), and OPVs (organic photovoltaic devices). So far, researchers have successfully designed and synthesized various novel BN-PAHs, including boron–nitrogen-doped benzene, naphthalene, anthracene, phenanthrene, pyrene, and polycyclic aromatic hydrocarbons with large conjugated structures. However, addressing stability issues under operational conditions is equally important for the practical deployment of devices based on BN PAH.

## 5. Application of BN-Doped Polycyclic Aromatic Hydrocarbons in Optoelectronics

### 5.1. Application of BN-Doped Polycyclic Aromatic Hydrocarbons in Optics

#### 5.1.1. Light-Emitting Devices

**Figure 4:**
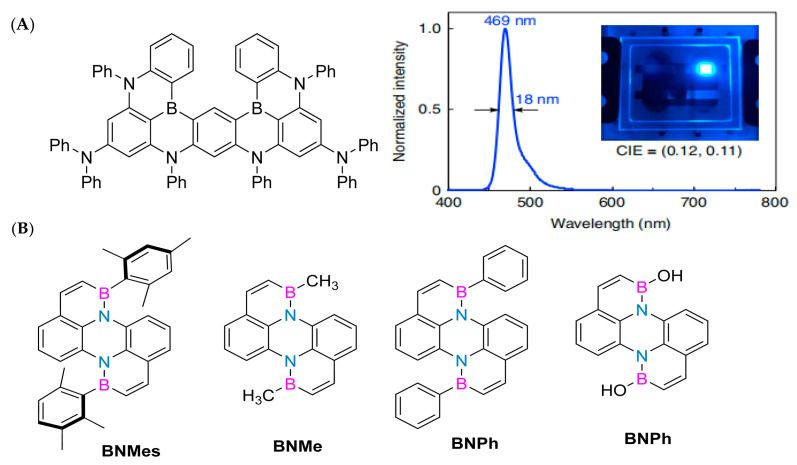
(**A**) Compound structure, electroluminescent spectra, and electroluminescent devices [57]. Reprinted with permission from Ref. [57]. Copyright 2019 Springer Nature. (**B**) Double boron–nitrogen heterocyclic aromatic hydrocarbons, including BNMes, BNMe, BNPh, and BNOH [61]. Reprinted with permission from Ref. [61]. Copyright 2022 John Wiley and Sons.

Due to their exceptional photophysical properties, molecules embedded with BN have demonstrated potential applications within the realm of optoelectronic devices. Specifically, in 2019, Hatakeyama’s research group innovatively conceptualized a novel polycyclic skeleton, ingeniously linking five benzene rings through two boron atoms, four nitrogen atoms, and two diphenylamino groups (Figure 4A). This distinctive design gives rise to multiple resonance effects between the boron and nitrogen atoms, leading to the distribution of the HOMO (highest occupied molecular orbital) and LUMO (lowest unoccupied molecular orbital) across different atoms. Consequently, the resultant non-bonded molecular orbitals (MOs) aid in minimizing vibrational coupling and relaxation within the material, thereby achieving remarkable performance with a photoluminescence (PL) band full width at half maximum of 14 nm. The organic light-emitting diode device prepared using this new emitter emits blue light at a wavelength of 469 nm, with a full width at half maximum of 18 nm and a maximum external quantum efficiency of 34.4%. At 1000 cd m^− 2^, the external quantum efficiency is 26.0% [57].

In 2021, the research group led by Chan in Japan successfully fabricated high-performance blue OLED devices by integrating the TADF material HDT-1 as the primary component, with the μ-DABNA blue light material serving as the donor, while leveraging the energy transfer mechanism between the two. The resulting device exhibited impressive performance parameters: a current efficiency reaching up to 39 cd/A, a full width at half maximum (FWHM) of merely 18 nm, and a color coordinate of CIE (0.15, 0.20). These outstanding performance indicators have set a benchmark for the subsequent development of more efficient and stable pure blue OLED devices [58]. Meanwhile, Duan’s group proposed a spectral red-shift strategy of “synergistic electron-coupling enhancement”. In this strategy, polycyclic aromatic hydrocarbons are doped with multiple B and N atoms, with B and N atoms arranged in an orderly manner around the central benzene ring in the form of B-π-B and N-π-N. This unique molecular structure enhances the push–pull electron effect within the molecule, allowing the emission spectra of these molecules to be extended to the deep red region. The corresponding deep red emitter with peaks at 662 and 692 nm exhibits a narrow full width at half maximum of 38 nm, a high radiation attenuation rate of approximately 108 s^−1^, and a 100% photoluminescence quantum yield. It is currently the reddest OLED device in MR-TADF molecules, with an external quantum efficiency (EQE) of up to 28%. This performance marks the highest level of performance of deep red OLED materials [59].

In 2022, the Wang team further expanded the material system by developing two polycyclic aromatic hydrocarbon (PAH) MR-TADF emitters (Cz BSN and DCz BSN) doped with boron (B), sulfur (S), and nitrogen (N). This innovative design combines the advantages of fast reverse intersystem crossing (RISC) of B and S-doped polycyclic aromatic hydrocarbons with the high photoluminescence quantum yield (PLQY) of B and N-doped polycyclic aromatic hydrocarbons. The optimized emitter DCz BSN achieves an 88% PLQY and a fast RISC rate of 1.0 × 10^5^ s^−1^. When applied to OLEDs, devices using DCz BSN emit narrowband blue light at 473 nm with a full width at half maximum (FWHM) of 29 nm and a maximum external quantum efficiency (EQE) of 22.0%, demonstrating competitiveness in narrowband blue light emission applications. This work not only enriches the structural library of MR-TADF emitters, but also provides a feasible method for adjusting emission characteristics through multi-heteroatom co-doping [60].

In 2023, Li Yuanhao successfully synthesized the key precursor II-24 through a series of chemical reactions, including a cross-coupling reaction, Wohl-Aue condensation reaction, reduction reaction, substitution reaction, and Stille coupling reaction. Subsequently, he further synthesized a class of polycyclic aromatic hydrocarbons with unique structures, including BNMes, BNMe, BNPh, and BNOH (Figure 4B), through an electrophilic boronization reaction. It is worth noting that BNMes exhibits particularly outstanding performance. Its fluorescence quantum yield is as high as 30.79% (Φ PL = 30.79%), and its thermal stability is excellent, with a temperature of up to 299 ° C at a weight loss of 95%. Given these excellent characteristics, BNMes has been further applied in the preparation of OLED devices. The experimental results show that when the voltage of the OLED device is 8 V, its brightness (Lmax) reaches 588.9 cd/m^2^. Under the condition of a current density of 12.55 mA cm^−1^, the maximum current efficiency (CEmax) of the device is 3.31 cd/A^−1^. When the current density is increased to 16.49 mA cm^−1^, its maximum power efficiency (PEmax) reaches 3.1 Lm/W^−1^. In addition, OLED devices based on BNMes also exhibit sky blue light emission characteristics, with color coordinates (CIE) of (0.1753, 0.3190). In summary, this new type of boron–nitrogen heterocyclic perylene compound has shown great potential for applications in the field of luminescence, opening up new directions for future research [61].

In recent years, significant progress has been made in the research of polycyclic aromatic hydrocarbons (PAHs) in the field of electronic materials. Among numerous PAHs, cyclic penta-fused polycyclic aromatic hydrocarbons (CP-PAHs) have attracted much attention due to their unique physical properties, especially their π-system and photophysical properties endowed by non-commutative electronic structures [62]. By replacing C-C units with B-N units, effective control of electronic structure has been achieved, leading to the development of a series of BN-doped CP PAHs with unique optoelectronic properties, such as BN-dibenzo [a,e] pentacene [41], BN-cyclohexene [63], BN-fluoroanthracene [44], and BN-dibenzo [a,m] rubicene [64]. Benzo [b] fluoroanthracene, as one of the representatives of CP-PAHs, can also be regarded as π-extended fluoroanthracene and has been studied by many researchers. In 2020, Shibata’s team introduced an efficient catalytic enantioselective synthesis method for benzo [b] fluoroanthracene-based axial chiral polycyclic aromatic hydrocarbons, which exhibited a high quantum yield and circularly polarized luminescence (CPL) characteristics [65]. In 2024, Su’s group proposed a direct strategy for constructing BN-doped benzo [b] fluoroanthracene and synthesized a series of BN-benzo [b] fluoroanthracene derivatives through a direct n-direction boronation cyclization pathway. These boronized derivatives exhibit significant electronic structure modifications. Of particular note is that BN-benzo [b] fluoroanthracene, containing amino functional groups, exhibits a unique blue emission characteristic, with a significant red shift in its spectrum compared to other target compounds. Further explanation through TD-DFT calculations shows that the introduction of amino groups is a key factor leading to the transition of emission behavior from the locally excited state (LE) to the charge transfer state (CT). In addition, based on BN-benzo [b] fluoroanthracene (specifically compound 2 with excellent properties) as the luminescent core, organic light-emitting diodes (OLEDs) were prepared. This practice not only verifies the feasibility of BN-benzo [b] fluoroanthracene as an OLED material but also indicates its potential application value in the field of optoelectronics. This series of research results not only enriches the understanding of the photophysical properties of boronized aromatic compounds but also provides strong experimental basis and theoretical support for the development of new organic optoelectronic materials [66].

In 2024, the compounds synthesized by Wu’s group exhibited distinct characteristics, including small Stokes shifts and narrow emission spectra that could be tuned across a spectrum ranging from deep blue light to orange-red light. Additionally, their super fluorescent OLED devices achieved an impressive external quantum efficiency of up to 40.1%, with a notable efficiency of 29.2% maintained at 1000 cd/m^2^. This research holds significant potential for advancing the commercialization of narrowband-emitting materials tailored for ultra-high-definition OLED displays. It also offers invaluable insights into the design and synthesis of novel organic optoelectronic materials, contributing to the ongoing advancement in this field [47].

#### 5.1.2. Light Detector

In 2022, Jin’s group successfully prepared five types of heterojunction photodetectors, which were obtained by doping P-type furan-fused boron–nitrogen aromatic hydrocarbons with N-type PC71BM in a ratio of 2:1. Their specific structure is ITO/PEDOT:PSS/FBNX:PC71BM/C60/LiF/Al. After a series of performance tests and analyses, the results show that compared with single-active-layer photodetectors, the performance of these heterojunction photodetectors is significantly improved. This improvement is mainly attributed to the intermolecular charge transfer state between the donor and acceptor materials within the heterojunction, which minimizes the π-π stacking of molecules and makes it more difficult for excitons to be captured during transport. Therefore, the dissociation rate of excitons and the mobility of charge carriers have been improved, resulting in strong photocurrent signals and excellent light detection capabilities [67].

Circularly polarized light (CPL) detection technology has received widespread attention and research in emerging fields such as optical communication, polarization imaging, and machine vision [68]. Due to the interaction between light and matter, CPL detectors constructed utilizing chiral materials possess the capability to directly and efficiently differentiate between left circularly polarized light (LCPL) and right circularly polarized light (RCPL), eliminating the necessity for intricate optical components. Notably, chiral organic semiconductor materials have demonstrated considerable promise in the realm of CPL detectors, exhibiting tunable detection wavelengths, ease of processing, and exceptional compatibility with flexible substrates. These attributes have sparked considerable interest among researchers. The pivotal parameters for assessing the performance of organic CPL detectors encompass the photocurrent asymmetry coefficient (g_ph_ = 2 (ILCPL-IRCPL)/(ILCPL+IRCPL)) and the detection wavelength. Despite substantial advancements in enhancing g_ph_, the majority of reported devices primarily exhibit a CPL response within the visible and near-infrared (NIR) regions, with organic UV-CPL detectors remaining relatively scarce [69]. The main obstacle in this field is the lack of chiral organic semiconductor materials that combine visible-light-blind UV-selective absorption with excellent charge transport performance.

**Figure 5:**
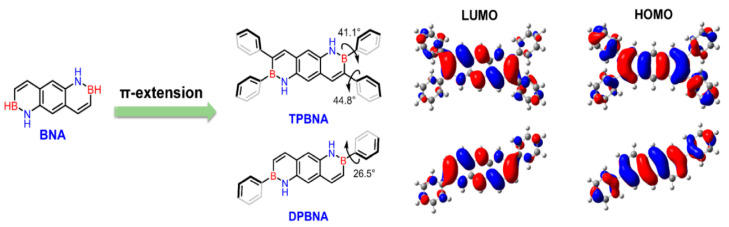
Periphery engineering of BN-PAHs to explore high-performance semiconductors [70]. Reprinted with permission from Ref. [70]. Copyright 2022 John Wiley and Sons.

In 2022, Wang’s group reported the incorporation of biphenyl-substituted BN units into anthracene, resulting in DPBNA, which exhibited exceptional performance in high-performance organic field-effect transistors and photodetectors. When compared to carbon-based electronic structures, such as diphenylanthracene (DPA), DPBNA was found to possess a wider energy gap between the highest occupied molecular orbital (HOMO) and the lowest unoccupied molecular orbital (LUMO) [70]. Therefore, in the solid state, DPBNA exhibits blue-shifted absorption characteristics, resulting in selective absorption of ultraviolet light. This selective absorption offers potential value for UV-CPL detection, particularly following the introduction of chiral elements (Figure 5).

Based on the above findings, Wang’s group further introduced chiral side chains into DPBNA in 2024, achieving the transfer of chirality from chiral molecules to aggregates while retaining the UV absorption properties of the DPBNA core [71]. BN-embedded anthracene derivatives with chiral 3,7-dimethyl side chains have emerged as a novel UV-CPL detection material. The research team constructed a p-n heterojunction-based ultraviolet circularly polarized light detection device (UV-CP-OPT), in which (S,S)- or (R,R)-DPNNA was used as the chiral photoactive layer, and the non-chiral n-type polymer F4BDOPV-2T (a NIR-absorbing conjugated polymer containing benzofuran diketone oligomer (BDOPV) units) was used as the electron transfer layer. The CP-OPT device achieved an average g_ph_ value of 0.033 when detecting 365 nm ultraviolet CPL, with higher g_ph_ values in the deep ultraviolet region. The experimental results show that CP-OPT devices have significant photoresponse and CPL resolution, which fully demonstrates the enormous potential of BN-embedded semiconductors as a new type of material in the field of UV-CPL detectors. The study demonstrates that by configuring heterojunction devices, both UV-selective absorption and efficient charge transfer can be achieved simultaneously, providing a universal strategy for designing UV-CPL detectors. In addition, it also provides a new type of BN-embedded organic semiconductor material for the development of future UV-CPL detection technology [72].

In 2024, Wu’s group successfully prepared a self-powered high-performance UV-A/B OPD based on the FBN-X heterojunction photosensitive layer. Five FBN-X compounds were doped into PC71BM as the heterojunction photosensitive layer to prepare a solution-treated high-performance organic photodetector (OPD) with a self-driven effect and high sensitivity to UV-A/B. Of particular note is that the FBN-CN-based device exhibits a detection rate of up to 1.72 × 10^12^ Jones, while the external quantum efficiency peak reaches 19.1%. Of particular importance is that the dark current density of the device is extremely low, only 8.90 × 10^−10^ A/cm^−2^, which fully demonstrates its high sensitivity to low-light detection. This is the first report of a photodiode-type UV-OPD containing BN-doped polycyclic aromatic hydrocarbons as the active layer. Compared with the reported 300–380 nm UV-OPD, the optimal device has significant advantages [73].

#### 5.1.3. Photocatalytic Material

Polycyclic aromatic hydrocarbon materials (BN-PAHs) containing B and N exhibit excellent optoelectronic properties as a novel π-conjugated system and have therefore received widespread attention.

**Figure 6:**
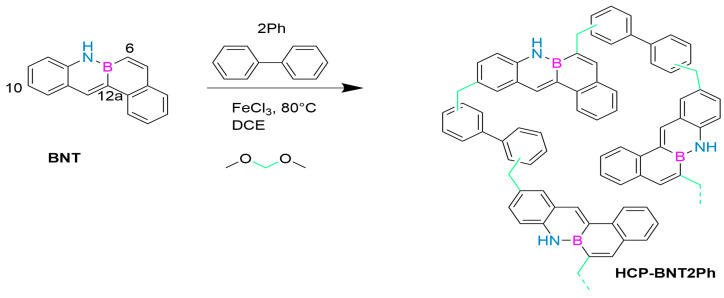
HCP-BNT2Ph [74]. Reprinted from Ref. [74].

In 2023, May’s group first reported a novel porous polymer containing B-N covalent bonds in its structure, which was prepared by reacting tetraphenyl-B-N monomer (BNT, Figure 6) with biphenyl (2Ph) as a comonomer. This synthesis strategy successfully produced the first member in the BN-PAH based super-crosslinked porous polymer series, HCP-BNT2Ph. It is prepared using a solvent weaving strategy, which allows the aromatic rings of two monomers to be connected through methylene provided by an external crosslinking agent. The polymer has a 98% performance in the nitrogen hetero Henry coupling reaction, a micro-mesoporous porosity, an SBET value of 612 m^2^/g, high thermal stability, and potential as a heterogeneous photocatalyst. After the first run, the catalyst improved its photocatalytic activity, reducing the reaction time to only 2 h and maintaining this activity during continuous operation. Research has shown that although the irradiation conditions are not the same, HCP-BNT2Ph is the fastest photocatalyst because it achieves complete conversion of THIQ in a shorter time (2–4 h) than the currently published coupling derivatives (10–448 h) and also achieves complete conversion of THIQ. The presence of free radicals in this structure remains stable during continuous operation. As a photocatalyst with high stability and efficient performance, it has shown broad potential for applications [74].

In 2024, Tian’s group successfully prepared a remote ordered layered assembly based on BN-PAH using the HSA strategy guided by synergistic cation-π and C-H···π interactions. By adjusting the solvent conditions in the two progressive assembly levels, the transformation process in which the one-dimensional structure gradually merges into a two-dimensional structure formed during the primary assembly process can be precisely controlled. This is mainly due to the careful design of the direction and binding motif of the two non-covalent forces, combined with the regulation of the self-assembly direction. In addition, the 2D-BNSA produced through the secondary assembly process has an ordered 2D layered structure and a specific “rigid yet flexible” assembly paradigm. This unique configuration enables it to effectively disperse and anchor various nanocatalysts, form heterostructures, enhance charge separation and transfer processes, and ultimately achieve efficient dual-function photocatalysis and electrocatalysis. Therefore, CdSe@2D-BNSA exhibits excellent photocatalytic efficiency in converting CO2 to CH4, with an evolution rate of 938.7 μmol g^−1^ h^−1^ for CH4. Ag NPs@2D-BNSA exhibits enhanced electrocatalytic performance in acetylene semi-hydrogenation, and the FE of ethylene can reach 98.5%. In summary, the study provides an effective strategy for precise control of the layered assembly process, structure, and function of BN-PAHs, thereby further exploring the use of BN-PAH-based superstructures as supramolecular multifunctional materials with multiple applications [75].

### 5.2. Application of BN-Doped Polycyclic Aromatic Hydrocarbons in Electronics

#### 5.2.1. Boron–Nitrogen Field-Effect Transistor

By incorporating boron (B) and nitrogen (N) atoms into the polycyclic aromatic hydrocarbon framework, a new type of material suitable for organic field-effect transistors (OFETs) can be designed. In 2011, Nakamura’s research group made a breakthrough by successfully synthesizing a class of heterocyclic molecules. Time-resolved microwave conductivity (TRMC) testing revealed that the hole mobility of these molecules is as high as 0.07 cm^2^∙V^−1^∙s^−1^, significantly increasing by an order of magnitude compared to their carbon skeleton analogues. This strongly demonstrates that BN-substituted polycyclic aromatic hydrocarbons (PAHs) are highly promising organic electronic materials [76]. In 2013, Pei’s group successfully prepared boron–nitrogen-substituted tetrathienonaphthalene molecules with different lengths using an optimized one-pot cyclization method. Theoretical calculations further confirm that the orbitals of boron–nitrogen atoms have achieved good delocalization throughout the entire π-conjugated system. The introduction of thiophene ring flattens the molecule and exhibits good thermal and chemical stability. In addition, the introduction of BN units promotes dipole–dipole interactions between molecules, resulting in a more ordered arrangement of molecules in a single crystal. Thin-film field-effect transistor devices based on these materials exhibit excellent hole mobility, reaching 0.15 cm^2^∙V^−1^∙s^−1^, marking the first application of boron–nitride-fused ring molecules in organic electronic devices and fully verifying the enormous potential of such organic semiconductor materials in the field of electronics [77].

**Figure 7:**
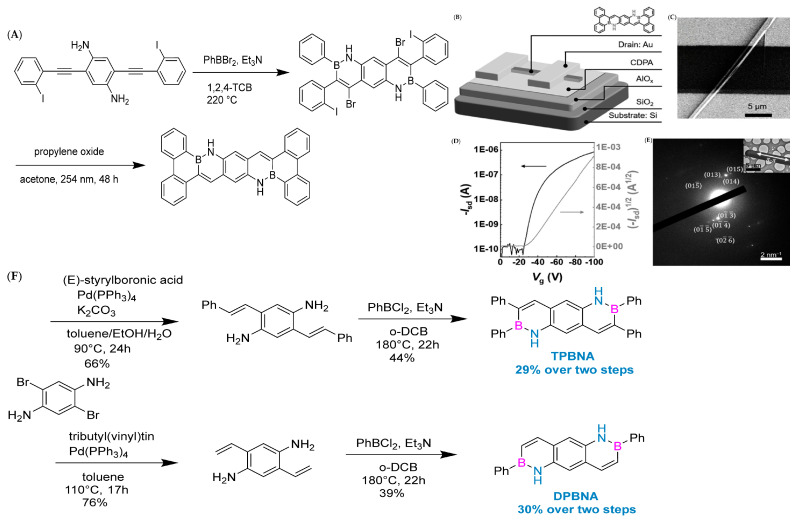
(**A**) Synthetic route to BNTBP [78]. (**B**) Architecture of the device [78]. (**C**) SEM image of the device [78]. (**D**) Transfer characteristics of the device. Isd=drain current, Vg = gatevoltage [78]. (**E**) SEAD pattern of the single crystal microribbon [78]. Reprinted with permission from Ref. [78]. Copyright 2019 John Wiley and Sons. (**F**) Synthetic routes to TPBNA and DPBNA [70]. Reprinted with permission from Ref. [70]. Copyright 2022 John Wiley and Sons.

In 2019, Pei’s group used the BN-fused ring synthesis strategy and integrated light-induced dehalogenation cyclization reaction to synthesize BN polycyclic aromatic hydrocarbons (Figure 7A). Subsequently, the band-shaped crystals extracted from dichloromethane and methane were used to construct FET (field-effect transistor) devices (Figure 7B–E). Furthermore, the line field-effect transistor device prepared based on single-crystal BNTBP exhibited a hole mobility of 0.33 cm^2^ V^−1^ s^−1^ [78].

In 2022, Pei’s group successfully proposed the first easily synthesized novel derivative of p-toluenediimide-B_2_N_2_-PDI. The structures of these compounds have been comprehensively validated through detailed characterization using nuclear magnetic resonance, mass spectrometry, and X-ray crystallography. Further research has revealed the significant effects of introducing BN units on the photophysical and electronic properties of B_2_N_2_-PDIs, which have been further understood through theoretical calculations. Compared with the parent compound, p-phenylenediimine (PDI), B_2_N_2_-PDI exhibits a deeper highest occupied molecular orbital energy level with new absorption peaks observed in the high-energy region. In addition, its absorption and emission maximum values exhibit a low color shift, and the photoluminescence quantum yield has decreased. The single-crystal field-effect transistor based on B_2_N_2_-PDI exhibits excellent performance, with an electron mobility of up to 0.35 cm^2^ V^−1^ s^−1^, fully demonstrating the potential application value of B_2_N_2_-PDIs in the field of optoelectronic materials [79].

In 2022, Wang’s group reported the implementation of high-mobility organic semiconductors based on existing BN-PAHs through a “peripheral engineering” strategy. They designed and synthesized BN anthracene substituted with tetraphenyl and diphenyl groups (TPBNA and DPBNA, respectively) (Figure 7C). Among organic field-effect transistors, DPBNA exhibits the highest hole mobility compared to TPBNA and all reported BN-PAHs, reaching 1.3 cm^2^ V^−1^ s^−1^. It is worth noting that this is the first BN-PAH with a mobility exceeding 1 cm^2^ V^−1^ s^−1^, which is the benchmark value for practical applications compared to amorphous silicon. Notably, it also marks the first successful demonstration of organic phototransistors based on DPBNA single crystals, which exhibit excellent photoresponsive performance. These research results not only mark an important technological breakthrough but also reveal the potential of BN-PAHs in the field of optoelectronic applications [70].

**Figure 8:**
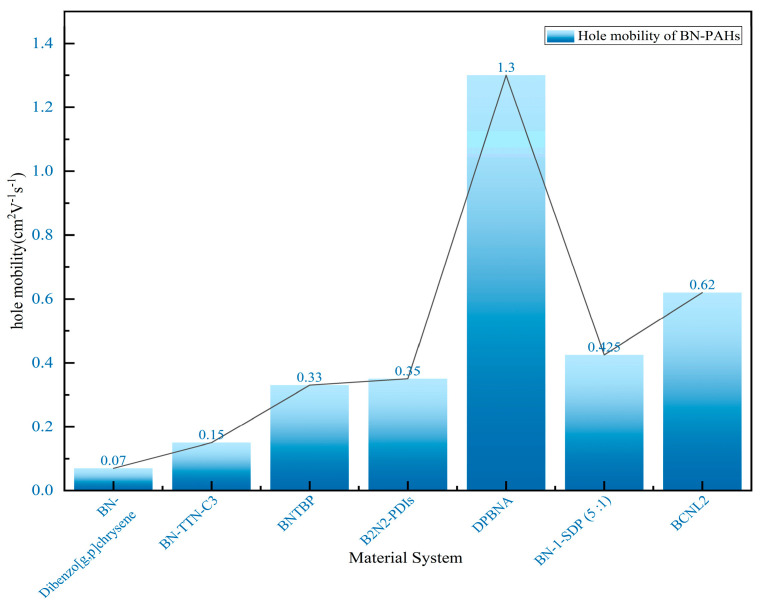
Hole mobility of BN-embedded PAHs in different material systems [70,76,77,78,79,80,81].

In 2023, Liu’s group synthesized two wide-bandgap U-shaped polycyclic aromatic hydrocarbons (BN-1 and C-1) doped with boron and nitrogen (BN) to meet the performance requirements of organic field-effect transistor non-volatile memory (OFET-NVM) and made corresponding adjustments to their electronic properties. Due to the electron donor effect of N and the high electron affinity of B, compared to C-1 and most reported small molecules, BN-1-based OFET-NVM exhibits a larger bipolar memory window and enhanced charge storage density. Moreover, the BN-1-SDP (5:1)-based device displays large and compact pentacene grains, leading to a high mobility of 0.425 cm^2^ V^−1^ s^−1^ (Figure 8). The new supramolecular system formed by BN-1 and PMMA helps to manufacture uniform thin films with a uniform microstructure, serving as a two-in-one tunnel dielectric and charge capture layer, achieving long-term charge retention and reliable durability. The research results indicate that both BN doping and supramolecular engineering are crucial for charge capture in OFET-NVM. This is the first time that BN-doped polycyclic aromatic hydrocarbons have been used as molecular floating gates to successfully manufacture high-performance non-volatile organic field-effect transistor (OFET) memory. Their work not only provides an effective method for synthesizing BN-doped polycyclic aromatic hydrocarbons but also elucidates the role of molecular design in charge capturing OFET memory [80].

The in-depth study of the charge transport properties of BN-PAHs still lags far behind the research on their emission performance. In 2024, Wang’s group reported the successful synthesis of novel stepped BN-PAHs (BCNL1 and BCNL2) with highly ordered BC_3_N_2_ acylene units, achieved through nitrogen-oriented tandem C-H boroylation. Through single-crystal X-ray diffraction analysis, their unique and compact herringbone filling structure was observed. In addition, the structure of micro single-crystal organic field-effect transistors (OFETs) helps enhance charge transfer capability. Compared to BCNL1, BCNL2 achieves a hole mobility of up to 0.62 cm^2^ V^−1^ s^−1^ (Figure 8), which is three orders of magnitude higher (μ_h_ max = 6 × 10^−4^ cm^2^ V^−1^ s^−1^ ), ranking as the highest value among BN-PAH-based OFETs. Detailed calculations attribute the significant increase in hole mobility to the notable decrease in the recombination energy (λ) of BCNL2, which is caused by the cyclization of the five-membered pyrrole ring and the elongation of the molecular skeleton. This provides insights into the molecular design principles of potential BN polycyclic aromatic hydrocarbons in optoelectronic applications [81].

#### 5.2.2. Photovoltaic Devices

In recent years, as a promising photovoltaic technology, organic solar cells (OSCs) have attracted much attention because of their advantages such as low cost, flexibility, translucency, non-toxicity and environmental protection, and support for large-scale roll-to-roll processing [82]. The energy conversion efficiency of organic solar cells continues to rise, which is inseparable from the emergence of efficient organic photovoltaic materials and the continuous development of device technology [83,84].

In 2016, Pei’s group synthesized a halobenzene aromatic hydrocarbon containing two boron–nitrogen units, which was innovatively applied to solution-processed solar cells, achieving a power conversion efficiency of 3.15%. This achievement marks the first application of borazine in the field of organic solar cells [85]. In the same year, Liu’s group also made significant progress, and they developed a series of polymer materials based on boron–nitrogen coordination bonds. Due to the existence of boron–nitrogen coordination bonds, the energy levels of these materials are significantly reduced, which makes them ideal acceptor materials. They have been successfully applied in the all-polymer heterojunction solar cell devices and have achieved high energy conversion efficiency [86,87,88].

In 2020, Pinheiro’s group designed a new type of high-efficiency SF sensitizer for solar cells, which provided useful guidance and a detailed understanding of how to use BN doping to fine-tune the exciton properties and chemical stability of Tetraene, as well as the chemical stability related to the characteristics of two bases. Considering the energy and exciton properties, among the 60 BN Tetraene systems studied, compounds 2,3, 5,18, and 18,2 may be the best SF sensitizers, because they show the most appropriate characteristics: the singlet state and the two triplet states have low external energy or a near-isoenergetic relationship, and the triplet exciton has ideal energy (ET1 1.0–1.3 eV), which can be compatible with the traditional charge absorption layer of solar cells. The exciton binding energy is small, and the chemical stability is good. It is worth noting that 2,3 and 5,18 BN Tetraene have a common feature, i.e., B and N dopants directly combine to form B-N bonds. These types of systems, also known as azaboron compounds, have been extensively studied and experimentally characterized, and synthesis technology has also made significant progress. The theoretical results are of great significance for the development of organic solar cell technology [89].

**Figure 9:**
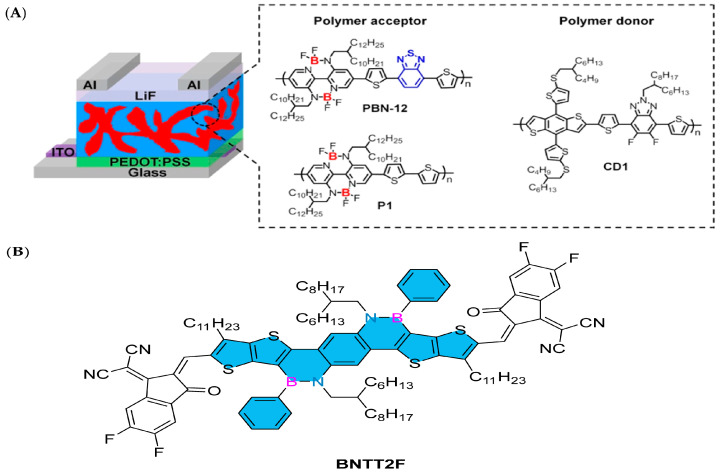
(**A**) The device structure of all-PSCs and the chemical structures of polymer acceptors (PBN-12 and P1) and a polymer donor (CD1) [91]. Reprinted with permission from Ref. [91]. Copyright 2020 American Chemical Society. (**B**) BNTT2F [94]. Reprinted with permission from Ref. [94]. Copyright 2023 CNKI (Beijing) Technology Co., Ltd.

Double B→N-bridged bipyridine (BNBP) has been used in the design of polymer receptors as an electron-withdrawing unit. In 2020, Liu’s group developed an organic boron polymer, which can be used as the acceptor material of all-polymer solar cells (all-PSCs), achieving a power conversion efficiency of up to 10.1%. The realization of this achievement is due to the incorporation of 2,1,3-benzothiadiazole units into the main chain structure of an organic boron polymer, which effectively adjusted its absorption spectrum, energy level structure, electron mobility, and phase separation behavior. All these improvements help to achieve excellent all-PSC device performance. In this study, the alternating copolymer (P1) of the BNBP unit and dithiophene unit was selected as the receptor of the control polymer [90]. By combining BT units into the backbone of P1, they obtained the target polymer receptor PBN-12 (Figure 9A). The influence of BT units on the photoelectric properties of the organic boron polymer and the performance of all-PSC devices has been systematically studied. These results show that the solution-processed polymer containing main group element atoms has excellent photoelectric device performance [91].

In 2021, Duan’s group reported a new monomer boron-azaarene BNT and its polymer PBNT-BDD. The core structure of this series of compounds is diboroazaanthracene. In the application of preparing high-efficiency organic solar cells (OSCs), when PBNT-BDD is mixed with the non-fullerene receptor Y6-BO, it shows excellent performance, and its power conversion efficiency (PCE) reaches 16.1%, which is equivalent to that of its benzo [1,2-b:4,5-b′] dithiophene (BDT)-based counterpart polymer. PBNT-BDD can effectively reduce the energy loss from singlet state to triplet state. The singlet–triplet energy gap (ΔEST) of PBNT-BDD is as low as 0.15 eV, which is much lower than that of ordinary organic semiconductors (≥0.6 eV). Therefore, the triplet state of PBNT-BDD is higher in energy than the charge transfer (CT) state, which will effectively inhibit recombination through the triplet state [92]. In 2022, the research group further reported an innovative acceptor–donor–acceptor (A-D-A)-type π-conjugated molecule based on boron-azaarene, named BNTT2F, which was designed to be used as an electron acceptor material in organic solar cells. The efficiency of the organic solar cell device constructed with BNTT2F has reached 8.3%, which once again strongly proves that boron-azaarene has broad development prospects in the field of organic solar cells [93]. The photoelectric conversion process of OSCs mainly occurs in the active layer. The innovation of active-layer materials, including new receptors and donors, has made a significant contribution to the improvement in the energy conversion efficiency (PCE) of OSCs.

In 2023, Liu’s group designed and synthesized a fused ring unit BNTT containing boron–nitrogen covalent bonds and connected it as an electron donor (D) unit with a strong electron-withdrawing (A) unit difluorocyanoindanone to prepare the A-D-A receptor molecule BNTT2F (Figure 9B). Thanks to the multiple resonance effect caused by the B-N covalent bond, BNTT2F shows a singlet–triplet energy gap (∆EST) as low as 0.20 eV, which is conducive to suppressing the energy recombination loss caused by the reverse transfer from the charge transfer state to the triplet state. An organic photovoltaic devices based on BNTT2F can achieve up to 8.3% PCE, which is also the highest efficiency of small-molecule receptors containing boron–nitrogen bonds. This shows that organic semiconductors based on boron–nitrogen-fused rings have great application prospects in the photovoltaic field [94].

#### 5.2.3. Electrochemical Sensor

Chemical sensors provide low-cost and portable detection through “naked eye” observation [95]. Chemical sensors with luminescent properties, with the help of various fluorescent technologies, can provide more spectral information, thus showing high selectivity and sensitivity [96]. The dual-channel sensor with colorimetric and fluorescent response is a very effective probe for detecting species in complex systems. Generally, boron-containing light sources are widely considered to be one of the most sensitive probes for detecting fluoride ions [97].

In 2010, Perepichka’s group made a breakthrough. For the first time, they successfully implemented the aromatic electrophilic substitution reaction on the thiophene ring, thus synthesizing a unique class of boron–nitrogen heterocyclic molecules. On this basis, Perepichka and his team further synthesized two boron aza oligothiophene structures. When n-Bu_4_NF was added to the solution of these compounds, the spectral properties of the molecules changed significantly: the absorption and emission spectra showed obvious red shift, accompanied by the emergence of new long-wave absorption and emission bands, while the original absorption and emission were weakened accordingly. Different from the absorption blue shift and fluorescence quenching phenomena of boron hetero-conjugated molecules in the detection of fluoride ions, the red-shift phenomenon in this system is more intuitive and more suitable for visual observation, so it has unique advantages in the detection of fluoride ions. Based on the fluorescence response characteristics of such molecules to fluoride ions, a new type of fluoride ion sensor is expected to be developed in the future [98].

In 2015, Zhang’s group successfully prepared a perylene imide-based boron-azaarene through an efficient intramolecular electrophilic boronization reaction, which was first reported as electron-deficient boron-azaarene in the literature. In-depth studies showed that this compound showed great potential as a highly sensitive and selective anion sensor. In the test of chloroform solutions with 12 different anions, only fluoride ions showed an obvious response, and the detection limit could be as low as 1.5 μmol/L [99].

**Figure 10:**
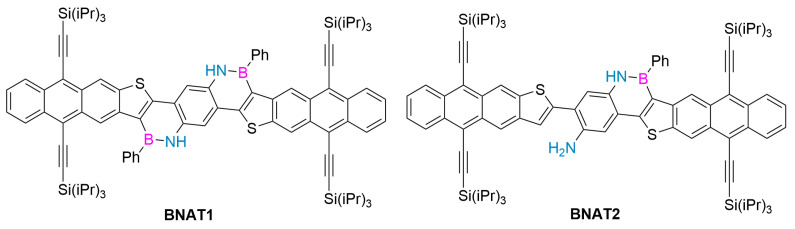
BNAT1 and BNAT2 [101]. Reprinted with permission from Ref. [101]. Copyright 2020 Elsevier.

In 2020, Wan’s group used scanning tunneling microscope (STM-BJ) technology and quantum transport calculations to study the single-molecule conductance properties of phenanthroline derivatives, their F^−^-coordination complexes, and corresponding C-C analogues. The introduction of B-N motifs leads to better single-molecule conductivity compared to C-C analogues. In addition, the Lewis acid–base reaction between F^−^ and the B atoms of the B-N motifs leads to a decrease in the conductivity of BN derivatives, which can be understood as the movement of the LUMO energy position, as revealed by quantum transport calculations, even if the HOMO-LUMO energy gap in the B-F Lewis acid–base decreases. Their work shows that the introduction of boron–nitrogen units into the framework of polycyclic aromatic hydrocarbons has become an effective strategy to regulate electron transport behavior. These findings provide valuable insights for adjusting the electron transport characteristics through the design of isoelectronic structures. B-N and other isoelectronic substitution structures are considered promising methods for the design of single-molecule devices such as switches and chemical sensors. This research achievement also opens up a new design idea for the future development of single-molecule devices such as chemical sensors [100]. In the same year, Wu’s group synthesized an anthracene [2,3-b] thiophene-based BN heteroarene with an 11-ring-fused backbone via a simple synthetic route, showing excellent air and light stability (Figure 10). The optical and electrochemical properties and DFT calculations of BNAT1 and BNAT2 were studied. The sensing ability of these compounds to fluorine was illustrated. BNAT1 can selectively detect fluoride due to the coexistence of BN units and triisopropyl groups, with an obvious color change and fluorescence quenching response. BNAT1 is more sensitive to visual detection of F^-^ than other related anions, indicating that BNAT1 is a promising F^−^ detector [101].

In 2021, Pei’s group designed and synthesized a novel BN aromatic hydrocarbon BN-Az with unique characteristics by combining boron–nitrogen-fused polycyclic aromatic hydrocarbons and azoene with strong intramolecular dipoles. They studied the structure, optical, electrochemical properties, and charge transport properties of BN-Az. It is worth noting that BN-Az exhibits significant selective color changes for fluoride ions and protons, which can also be monitored through nuclear magnetic resonance spectroscopy and single-crystal X-ray analysis, indicating its potential as an effective ion sensing material in stimulus-responsive electronic devices [102]. In the same year, Professor Wu Di and his colleagues designed and synthesized a heteroaromatic hydrocarbon with nine fused rings (ADTBN) and two trialkylsilylacetylene groups which exhibited good stability and solubility. A comprehensive study was conducted on the optoelectronic properties and fluorination rate of ADTBN. ADTBN exhibits selective and sensitive dual-channel fluorescence quenching towards fluoride anions, and they revealed that it may be a potential candidate for sensors [103].

In 2023, Huang’s research group conducted a comprehensive analysis of two different splicing modes of BN pentacyclic isomers (P-BN-BT and I-BN-BT), and found that their geometric structure, aromaticity, and photophysical properties did change with the variation in the splicing model. More importantly, P-BN-BT showed strong sensitivity to 2,4,6-trinitrophenol (TNP). The visual inspection of TNP can be easily carried out by making test strips, reaching the ppm level under sunlight. This discovery has greatly expanded our understanding of the structure and function of boron–nitrogen-doped polycyclic aromatic hydrocarbons, making them very promising in the field of chemical sensors in the future [104].The comparison of performance indicators of BN-PAH based fluoride ion sensors mentioned above is shown in the table below (Table 1).

## 6. Conclusions

This paper systematically reviews the latest application progress of BN compounds in the optoelectronic field in recent years, including light-emitting devices, photodetectors, photocatalytic materials, field-effect transistors, photovoltaic devices, electrochemical sensors, and other optoelectronic applications. In particular, significant progress has been made in the field of organic field-effect transistor (OFETs), organic light-emitting diodes (OLEDs), and organic photovoltaic devices (OPVs). A new type of organic semiconductor material with unique properties emerged by using isoelectronic BN units instead of C-C units in organic p-systems. This BN substitution strategy can not only effectively control the photophysical properties and redox properties of conjugated molecules, but also significantly affects the interaction between molecules. This discovery opens up a new research direction for the application of BN-embedded polycyclic aromatic hydrocarbons in optoelectronic devices. This work is expected to deepen our understanding of the electronic and optical properties of BN-containing aromatic hydrocarbons and provide a new method for the design of polycyclic aromatic hydrocarbons containing BN intercalation to further expand their photoelectric applications.

Although nitrogen heterocyclic compounds have shown potential applications in some fields, the practical application of BN-intercalated polycyclic aromatic hydrocarbons in optoelectronic devices is still in its infancy. Specifically, compared with similar materials, device performance needs to be improved, as there is still a certain performance gap. The rapid development of this field is facing multiple challenges, including poor material accessibility caused by the difficulty of synthesis and insufficient thermal and chemical stability. Therefore, there is an urgent need to explore efficient synthesis strategies to enrich the chemical diversity of BN-containing compounds. In addition, it is important to explore the internal relationship between BN-substituted aromatic structures and device performance to promote the development of this field. It can be predicted that the rational design of BN-substituted polycyclic aromatic hydrocarbons is expected to bring new opportunities for high performance and unique functions of organic electronic products, so as to further expand their application range.

Although the application of boron–nitrogen heterocyclic molecules in the field of organic electronics is still in the preliminary stage, they have initially demonstrated excellent device performance. However, the type and quantity of such materials are relatively scarce, which undoubtedly increases the difficulty of exploring the relationship between structure and performance. Although theoretical chemistry methods provide a way to predict the orbital and related properties of boron–nitrogen heterocyclic molecules, it is still necessary to synthesize a large number of molecules with different structures to summarize the specific effects of the covalent insertion of BN units on the electronic properties of organic π-conjugated materials. After nearly 60 years of continuous exploration, the synthesis of boron-aza aromatic fused ring compounds is still a major challenge in the field of organic chemical synthesis. Although the electrophilic substitution reaction has been widely adopted as a common method, the method of covalently embedding BN units into conjugated structures is still scarce. Although new synthetic routes have been proposed during this period, most of these methods have a limited scope of application and it is difficult to widely popularize them. These difficulties in synthesis undoubtedly hinder the further development of boron–nitrogen heterocyclic molecules in the field of organic electronics. Therefore, the continuous development of new strategies for the efficient synthesis of boron aza aromatic fused ring compounds is of great significance to promote the research and application of such materials. This not only helps to expand the variety and quantity of materials but also lays a solid foundation for further exploring the relationship between structure and performance.

## Figures and Tables

**Table 1:** Comparison of performance indicators of BN PAH-based fluoride ion sensors [97,98,99,100,101,102,103,104].

Specific Compounds/Materials	Limit of Detection (LOD)	Selectivity	Stability
PDI—1BN (Single BN-Fused Perylene Diimide)	1.5 μM (in CHCl_3_)	Only responds to F^−^ (among 12 anions, only F^−^ induces fluorescence quenching/absorption change; Ac^−^ has only slight response at 100 equiv)	Thermal decomposition temperature of 402 °C (TGA); stable in solid state at room temperature, soluble in CHCl_3_/THF
1a/1b (Azaboraindacenoheterole)	F^−^ not directly measured; logKa = 3.3 (F^−^ binding constant in CH_2_Cl_2_)	Only responds to F^−^ (no interaction with H_2_O, THF, Cl^−^, Br^−^, etc.)	Thermal stability ~300 °C (TGA); stable in solid/solution state (duration not specified); stable under nitrogen but oxidizes in air.
1,5,9—Triaza—2,6,10—triphenylborane (Crown—ether Analogue)	F^−^ not directly measured; stepwise hydroxylation response in wet solvent	Not directly measured for F^−^; the B–Ph bond mainly reacts with H_2_O/OH^−^, indirectly reflecting insufficient selectivity for F^−^	Thermal decomposition temperature of 322 °C (under inert atmosphere); soluble in chlorinated solvents; easily degrades in moist solvents (generating byproduct 4 within 14 days)
BN—substituted Phenanthrene Derivative (Single-Molecule Junction)	F^−^ not directly measured; 4-fold decrease in conductance at 1 eq F^−^	Only responds to F^−^ (C = C analogues have no response to F^−^)	Stable in solution (CH_2_Cl_2_); reversible conductance response in single-molecule junction state
BNAT1 (Eleven-Ring Fused BN Polyarene)	3.85 μM (in THF)	Only responds to F^−^ (not affected by Cl^−^, Br^−^, OAc^−^ etc.)	Stable for several months in solid/solution (THF); good thermal stability
BN—Az (BN-Fused Diazo—Carbazole)	F^−^ not directly measured; color development at 1 eq F^−^	Only responds to F^−^ (no interaction with Cl^−^, I^−^, NO_3_^−^, etc.); reversible response to protons (TFA)	Stable at room temperature in solid state; reversible Lewis acid–base reaction in solution (THF) (slight decrease in sensitivity after several cycles)
entry ADTBN (Nine-Ring Fused BN Polyarene)	F^−^ not directly measured; saturated response at 5 equiv TBAF	It is highly specific for F^−^, (not affected by Cl^−^, Br^−^, SO_4_^2−^, etc.)	Solid stable for several months at room temperature, soluble in THF/CH_2_Cl_2_
P-BN-BT	130 nM (TNP-related fluorescence quenching, F^−^ not directly measured)	F^−^ not directly measured; resistant to TNP interference (not affected by 2,4-dinitrophenol, etc.)	Solid white, soluble in chlorinated solvents, stable at room temperature (duration not specified, presumably several months)

## Data Availability

No new data were created or analyzed in this study. Data sharing is not applicable to this article.
